# Episomal Vectors for Stable Production of Recombinant Proteins and Engineered Antibodies

**DOI:** 10.3390/antib13010018

**Published:** 2024-03-11

**Authors:** Ian Fallahee, Daniel Hawiger

**Affiliations:** Department of Molecular Microbiology and Immunology, Saint Louis University School of Medicine, St. Louis, MO 63104, USA

**Keywords:** sortase, srt4m, recombinant chimeric antibodies, bispecific antibodies, intein, antibody–oligonucleotide conjugate

## Abstract

There is tremendous interest in the production of recombinant proteins, particularly bispecific antibodies and antibody–drug conjugates for research and therapeutic use. Here, we demonstrate a highly versatile plasmid system that allows the rapid generation of stable Expi293 cell pools by episomal retention of transfected DNA. By linking protein expression to puromycin resistance through an attenuated internal ribosome entry site, we achieve stable cell pools producing proteins of interest. In addition, split intein–split puromycin-mediated selection of two separate protein expression cassettes allows the stable production of bispecific antibody-like molecules or antibodies with distinct C-terminal heavy chain modifications, such as an antigen on one chain and a sortase tag on the other chain. We also use this novel expression system to generate stable Expi293 cell pools that secrete sortase A Δ59 variant Srt4M. Using these reagents, we prepared a site-specific drug-to-antibody ratio of 1 antibody–siRNA conjugate. We anticipate the simple, robust, and rapid stable protein expression systems described here being useful for a wide variety of applications.

## 1. Introduction

Recombinant engineered antibodies have become commonplace in modern medicine [1,2] and are typically produced by transient transfection for research purposes. For large scale biotherapeutic manufacturing, Chinese Hamster Ovary (CHO) cells are dominant, but there are increasing examples of Human Embryonic Kidney (HEK) 293-based cells being used for the production of FDA-approved therapeutics [3]. While production in CHO cells is suitable for standard antibodies with limited glycosylation [4,5], more complex molecules, such as protein–Fc fusions, may contain non-human carbohydrates such as galactose-α-1,3-galactose [6] and N-glycolylneuraminic acid [7]. Therefore, this warrants the further development of human-derived expression systems, which eliminate the risk of specific allergic responses and anaphylactic reactions stemming from the immunogenicity of these non-human carbohydrates [6,7,8]. HEK293-based cell lines are widely used in protein production for research [9] and the availability of current good manufacturing practices (cGMP)-banked Expi293F lines [10] affords the potential to transition to commercial production. Therefore, methods that simplify and accelerate the stable production of proteins in HEK293-based cells are of significant interest.

Stable protein production typically involves the integration of the transgene of interest into the cell line’s genome, a random and low probability event following the transfection of linearized DNA [11]. More efficient integration can be achieved by lentivirus transduction [12] or the co-transfection of the transgene of interest (flanked by inverted terminal repeats) with a transposase plasmid [13,14,15,16]. Here, we demonstrate a simpler approach using episomal vectors that allows the rapid generation of stably-transfected Expi293 cell pools without the need for lentiviral transduction or exogenous transposons.

Scaffold/matrix attachment region (S/MAR) elements and replication initiation (IR) elements have previously been used in vectors for recombinant protein production [17,18,19] or for the gene therapy of human cells [20]. S/MAR elements contain sequences that serve as binding sites for proteins such as scaffold attachment factor A, which tethers these elements to the nuclear matrix [21]. IR elements act as a mammalian origin of replication, initiating DNA replication with each cell division [22]. Depending on the particular arrangement of S/MAR and IR elements, plasmids can be integrated and amplified in the genome as a result of double-stranded (ds) DNA breaks [23] or they can be maintained episomally without integration or gene amplification [20,24]. To avoid the disruption of the expressed gene of interest by random DNA breaks [25], we chose an episomal design of S/MAR and IR elements previously established by Stavrou et al. [20,26]. This design was shown to allow the long-term replication of the introduced plasmid once per cell cycle in human hematopoietic progenitor cells [20].

As episomal vectors do not undergo gene amplification, we chose an alternative way to increase protein production. Within these vectors, we coupled protein expression to puromycin resistance (PuroR) by an attenuated internal ribosome entry site (IRES) [27]. An attenuated IRES reduces the expression of the PuroR protein relative to the protein of interest, allowing for increased selection stringency that results in higher producing cells [27,28]. This linked PuroR approach is additionally beneficial for long-term pool stability as the coupling of the expression of the protein and selectable marker to a single promoter reduces the chances of the selective silencing of the protein of interest [28,29]. Here, we show that episomal retention combined with protein-linked attenuated expression of PuroR allows the rapid generation of stable Expi293F pools producing the recombinant proteins of interest. Although it is theoretically possible for rare integration events to occur with episomal plasmids, a rare integration event should not harm the ability to express protein as long as the selection cassette is not expressed independently of the protein cassette (the chances of which are minimized by the use of IRES).

One of the recombinant proteins we produced by secretion into supernatants was Sortase A Δ59 variant P94S/D160N/D165A/K196T (Srt4M) [30,31], a highly efficient bioconjugation enzyme [30]. The secretion of recombinant proteins in mammalian cells, especially those not naturally secreted, is a non-trivial task [32]. Attempts have been made to generate synthetic signal peptides based on machine learning [32,33,34], but these methods are not yet fully reliable. Therefore, rather than use a synthetic signal peptide, we took inspiration from the findings of Güler-Gane et al. [32] by using natural mammalian signal peptides with predicted cleavage that matches at least the +1/+2 residues of the protein of interest. This strategy revealed several novel signal peptides that enable the secretion of Srt4M in mammalian cells.

The main class of recombinant proteins we focused on was engineered antibodies. Using the attenuated PuroR expression approach, we demonstrated the stable expression of engineered antibodies with C-terminal antigens or tags. C-terminally modified antibodies are typically used for sensitive immunological studies in mice [35,36,37,38] and cancer immunotherapy in humans [39,40]. However, when it comes to producing bispecific antibody-like molecules or antibodies with distinct C-terminal modifications on each chain, at least two different expression cassettes are required. By splitting the PuroR protein using a recently described split intein system [41], we efficiently selected for two separate expression cassettes. Specifically, we made use of the SiMPl split intein–split puromycin system, described by Palanisamy et al. [41], to produce stable Expi293 cell pools expressing a bispecific antibody–protein–Fc fusion and antibodies with multiple distinct heavy chain C-terminal tags, including a single sortase conjugation site. Ultimately, we demonstrated the applicability of these new approaches by preparing a site-specific drug-to-antibody ratio of 1 (DAR1) antibody–siRNA conjugate by Srt4M-mediated ligation and click chemistry [42].

## 2. Materials and Methods

All plasmid vector backbones are available from Addgene. The tricistronic EGFP-2A-PuroR vector backbone is available at Addgene ID 208377. The tricistronic N-terminal-PuroR-N-terminal-intein vector backbone is available at Addgene ID 208378. The tricistronic C-terminal-PuroR-C-terminal-intein vector backbone is available at Addgene ID 208379.

### 2.1. Vector Construction

The original backbone CMV expression vector was obtained from Twist Bioscience (South San Francisco, CA, USA). Whenever possible, sequences were ordered from Twist Bioscience or Integrated DNA Technologies (Newark, NJ, USA). All sequences were codon-optimized for expression in human cells using the built-in codon optimizer tools from each company. Further, all ordered sequences excluded restriction enzymes NotI, EcoRI, BstXI, NheI, XmaI, SalI, XbaI, FseI, AscI, SbfI, and MluI except at their designated positions, to maintain flexibility of the plasmids. For sequences that could not be synthesized due to repeated nucleotides or other internal complexities such as EMCV IRES, β-globin IR, and beta-interferon S/MAR, they were PCR-amplified using Accuprime Pfx (12344024 Invitrogen/ThermoFisher, Waltham, MA, USA) as follows: EMCV IRES was amplified from plasmid pEP4 E02S EN2L [43], which was a gift from James Thomson (Addgene plasmid #20922; http://n2t.net/addgene:20922; RRID:Addgene_80391). β-globin IR was amplified from BAC clone RP11-1205H24 obtained from BACPAC Genomics Inc. (Redmond, WA, USA). β-interferon S/MAR was amplified from plasmid pBMN(CMV-copGFP-Puro-SMAR) [44], which was a gift from Magnus Essand (Addgene plasmid #80391; http://n2t.net/addgene:80391; RRID:Addgene_80391). The split puromycin–intein sequences were synthesized by Twist Bioscience based on the sequences in Addgene plasmids 134318 and 134319 [41].

### 2.2. Cloning of Single Proteins (e.g., Srt4M) Plasmids

The EGFP-P2A-PuroR vector backbone contains unique restriction sites for cloning by restriction enzyme digestion and ligation (Figure S1A). For cloning of single proteins such as Srt4M, we ordered a DNA fragment encoding Srt4M with upstream NotI (underlined) and Kozak sequence before the start codon “gcggccgcgccgccaccatg” with an NheI site added after the stop codon. The insert and the EGFP-P2A-PuroR vector backbone (Figure S1A) were digested with NotI and NheI, appropriate fragments were gel purified, ligated together for 30 min at room temperature according to standard NEB ligation protocol, and used to transform DH5α or NEB Stable.

### 2.3. Cloning of Antibody Plasmids

Antibody vectors are cloned by a 4-part ligation including light chain, WT IRES, heavy chain, and the vector backbone. The light chain sequence was ordered with upstream NotI (underlined) and Kozak before the start codon “gcggccgcgccgccaccatg” with an EcoRI site added after the stop codon. The beginning of the heavy chain sequence was ordered in frame with a BstXI site as ccacaaccatgg (underlined atg is the heavy chain signal peptide start codon) with an NheI site added after the stop codon. The WT IRES fragment (between EcoRI and BstXI sites) was obtained by digesting any of the vector backbones. The desired vector backbone, either EGFP-P2A-PuroR (Figure S1A), N-terminal intein or C-terminal intein plasmid (Figure S2A), was then digested with NotI and NheI. The light and heavy chains were digested with corresponding enzymes as described above. All 4 gel-purified fragments (light chain, heavy chain, IRES, and vector backbone) were ligated together at room temperature for 2 h according to standard NEB protocols and used to transform DH5α or NEB Stable.

### 2.4. Single Plasmid for Bispecific Production

To obtain a single vector, we digested the N-terminal intein plasmid with MluI and SbfI to obtain the vector backbone after gel purification. We also digested the C-terminal intein plasmid with MluI and SbfI to excise the expression cassette that was also gel purified. We ligated both fragments together for 30 min at room temperature using standard NEB protocols and used the ligation mixture to transform NEB Stable (30 °C). This allows the efficient formation of a combined vector containing both tricistronic cassettes linked to N- and C-terminal intein–puromycin cistrons, with each cassette containing an S/MAR and the entire plasmid containing a single IR. This combined vector loses many unique restriction sites and therefore cannot easily be used for further cloning. It is instead used directly for transfection as a single plasmid containing both the N-terminal tricistronic cassette and the C-terminal tricistronic cassette.

### 2.5. Cells, Transfection, and Selection

MutuDC1940 [45] were obtained from Applied Biological Materials Inc. (Cat. No. T0528, Vancouver, BC, Canada).

Expi293F cells (A14527 Gibco/ThermoFisher, Waltham, MA, USA) were grown in a humidified 8% CO2 incubator. For culture volumes of 20 mL or more, cells were shaken at 125 rpm. For smaller culture volumes of 10 mL or less, cells were shaken at an angle at speeds between 125–145 rpm in 50 mL bioreactor tubes. Cell viability and density were measured using a Countess II FL Automated Cell Counter (ThermoFisher, Waltham, MA, USA). DNA for transfection was purified using EndoFree Plasmid Maxi Kit (Qiagen, Hilden, Germany). For single plasmid transfections, we used 75 μg DNA per 60 × 10^6^ cells at a density of 20 × 10^6^ cells/mL with a polyethylenimine (PEI)/DNA ratio of 3:1 and 0.1% Pluronic F-68 in Expi293 media for 3 h as described in Fang et al. [46]. As also described in Fang et al., more DNA can be used but PEI should be kept at 225 μg per 60 × 10^6^ cells. For co-transfection of two plasmids, we used 375 μg of DNA per 200 × 10^6^ cells, split between the two plasmids (187.5 μg DNA/200 × 10^6^ cells per bispecific plasmid) while keeping PEI at 750 μg per 200 × 10^6^ cells. After transfection, cells were diluted to approximately 1 × 10^6^ cells/mL, typically in an equal mixture of Expi293 media (A1435101, Gibco/ThermFisher, Waltham, MA, USA and EX-CELL 293 media (14571C MilliporeSigma, St. Louis, MO, USA). Expi293 media can be used for the entire process; however, to reduce media expenditures, we typically cultured cells in a mixture of both media during selection and eventually adapted them to grow only in EX-CELL 293 media (after 3–4 weeks). Puromycin (ant-pr-1 InvivoGen, San Diego, CA, USA) was typically added at 2–6 μg/mL 24–48 h after transfection. During the first month after transfection, the puromycin concentration was sometimes kept at 2–6 μg/mL and was sometimes adjusted based on cell viability and density. Typically, 2 μg/mL is sufficient to generate stable pools, but we tended to increase selection pressure as cells continued to expand. When cell density was above 1 × 10^6^ cells/mL, higher concentrations of puromycin were temporarily used (typically 20–60 μg/mL) compared to when cell density dropped below 1 × 10^6^/mL. When cell density dropped below 3 × 10^5^ cells/mL, 0–2 μg/mL puromycin was temporarily used (from 12 h to a few days) until the cells recovered, and then higher dose selection was resumed. If selection is removed for too long, low-dose resistance to puromycin can emerge. This can be resolved either by increasing the dose of selection or sorting for GFP^+^ cells. We defined a cell pool as stable at a particular dose of puromycin when after seeding at 3–5 × 10^5^ cells/mL into fresh selective media, cells doubled approximately every 24 h while maintaining > 95% viability without changes in mean GFP intensity. Selection with puromycin was maintained during regular passaging, however, before final production, selection was removed for at least 2 passages to ensure the least stress on the cells.

### 2.6. Antibody Expression and Protein Purification

For protein expression, cells were seeded at a density of 1.5 × 10^6^ cells/mL, typically in EX-CELL 293 media (though Expi293 media supports higher cell densities/yields) and valproic acid was added to the culture at a concentration of 3.5 mM as described in Fang et al. [46]. After about 5 days (or when viability dropped below 50%), supernatants were spun down successively at 250 g, 1600 g, and 4600 g for 30 min followed by sterile filtering with a 0.22 µm filter. Filtered supernatants were applied directly onto a column. Alternatively, large volumes could be further concentrated and dialyzed into DPBS using a Vivaflow 50R crossflow cassette (Sartorius, Göttingen, Germany) before column purification. Antibodies were purified by gravity column using Protein G Sepharose 4 Fast Flow Resin (Cytiva, Marlborough, MA, USA) and proteins containing His tags were purified on Ni-NTA Agarose (R90101 Invitrogen/ThermoFisher, Waltham, MA, USA).

### 2.7. Sortase Signal Peptide Library Construction

The entire secreted mammalian proteome was downloaded from UniProt, and proteins including signal peptides were sorted based on the first two amino acids after the predicted signal peptide cleavage site (according to UniProt). Peptides from proteins with QA sequence (matching the QA of Srt4M Δ59) were initially selected and further curated manually to obtain 17 signal peptides that were chosen to be part of a signal peptide library (Table S1). Three DNA fragments were ordered from Twist Bioscience that each contained sequences of 5–6 signal peptides along with in-frame restriction enzyme sites (NotI and BlpI). Each fragment was digested with NotI and BlpI and the mixture of individual DNA fragments encoding signal peptides was ligated into the plasmid with Srt4M described above and used to transform DH5α. For each of the three ligations (with no visible control ligation colonies), around 40 colonies were selected, grown together in liquid culture, purified by EndoFree Plasmid Maxi kit (Qiagen, Hilden, Germany), and then transfected into Expi293F cells. Puromycin (ant-pr-1 Invivogen, San Diego, CA, USA) was added after the first 48 h. When cell density was above 1 × 10^6^ cells/mL, higher concentrations of puromycin were used (60–100 μg/mL) compared to when cell density dropped below 1 × 10^6^/mL. When cell density dropped below 3 × 10^5^ cells/mL, 0–2 μg/mL was temporarily used until the cells recovered. After the first month, 20 μg/mL puromycin was used. For deglycosylation experiments, PNGase F (P0710S New England Bioabs, Ipswich, MA, USA) was used. For western blot to detect His_6_ tag, we used HRP anti-His tag antibody (652503 BioLegend, San Diego, CA, USA).

### 2.8. Signal Peptide Library PCR Amplification and Sequencing

Cells were incubated at 52 °C in Proteinase K-containing detergent buffer. PCR primers (F primer 5′ agatcagatctttgtcgatcctacca 3′ and R primer 5′tcatctcccggggttgtggc 3′) were used to amplify the entire first cistron and IRES using Accuprime Pfx (12344024 Invitrogen/ThermoFisher, Waltham, MA, USA) for 30 cycles. The amplified fragments corresponding to the expected MW were gel purified and sent to Plasmidsaurus (Eugene, OR, USA) for standard Oxford Nanopore sequencing of linear fragments. Only complete reads including the entire signal peptide to the beginning of Sortase A without errors were considered in the analysis.

### 2.9. Antibody and Protein–Fc Constructs

αDEC-205 constructs were previously described in Hawiger et al. [35]. αDEC-205-OVA and MOG sequences at C-terminus with linkers are as previously described [47,48]. αDEC-205-OVA-LPETGH_6_ included LPETGH_6_ in place of MOG or OVA antigens. αDEC-205 with a single sortase tag included LPETGH_6_ directly fused to the C-terminus of one heavy chain without any linker. For bispecific αDEC-205-mouse PD-1-Fc, a mutant IgG3 linker was used (IgG3C-) [49] with two repeats (ELKSPRSPEPKSSDTPPPSPRSPEPKSSDTPPPCPRCPAPEL). An Ig light chain signal peptide (MAWTPLLLPFLTLCIGSVVS) was used to express mouse PD-1 residues 25–150 (C83S) with (ERILELK) as the overlap to the IgG3 hinge. For deglycosylation experiments, deglycosylation mix II (P6044S) was obtained from New England Biolabs (Ipswich, MA, USA). Some deglycosylation enzymes can be seen on SDS-PAGE gels when added. PE/Cyanine7 anti-mouse CD205 (DEC-205) Antibody was purchased from BioLegend (138209, San Diego, CA, USA).

### 2.10. siRNA

The siRNA was purchased from TriLink Biotechnologies (San Diego, CA, USA) with HPLC purification. The sequences were based on Katakowski et al. [50] but with additional modifications as described below. The antisense siRNA sequence that was ordered was as follows: 5′ [Phos(H)] A (ps) [fA] (ps) G [fU] C [fG] U [fA] G [fA] G [fU] C [fC] A [fG] U [fU] G (ps) U (ps) U 3′ where all bases were 2′ O-Methyl modified unless indicated in brackets, [fX] indicates 2′ Fluoro Base, [Phos(H)] indicates 5′ Phosphate, and (ps) indicates phosphorothioate bond. The sense siRNA strand was as follows: 5′ C (ps) A (ps) [fA] C [fU] G [fG] A [fC] U [fC] U [fA] C [fG] A [fC] U U [SSC6] 3′ where all bases were 2′ O-Methyl modified by default unless indicated in brackets, [fX] indicates 2′ Fluoro Base, (ps) indicates phosphorothioate bond, and [SSC6] indicates 3′ C6 Disulfide Linker. Individual siRNA strands were dissolved in H_2_O at approximately 6 μg/μL. They were then mixed together equally and annealed by heating to 90 °C for 1 min, then placed in a 70 °C water bath and allowed to cool gradually over an hour with the water bath turned off. After annealing, siRNA was treated with excess TCEP (to reduce the C6 disulfide linker) and then buffer-exchanged back into water using a Pierce Protein concentrator with 3K MWCO (88512 ThermoFisher, Waltham, MA, USA).

### 2.11. Antibody–siRNA Conjugation

Approximately 56 μg of Srt4M (freshly buffer exchanged into DPBS to remove TCEP that is contained in the Srt4M stock as described in Li et al. [30]) was added to 150 μg of antibody with excess GGG-PEG_4_-DBCO and 5 mM CaCl_2_ in a total volume of approximately 200 μL. After 15 min, 20 mM EDTA was added to quench the reaction, and the antibody, Srt4M, and excess GGG-PEG_4_-DBCO were separated by SEC using an ENrich SEC 650 column (7801650 BioRad, Hercules, CA, USA) on a BioRad NGC Quest 10 Plus (7880003). Azido-PEG_3_-Maleimide was prepared according to the manufacturer’s protocol under Argon gas in dry DMSO. Azido-PEG_3_-Maleimide (in DMSO) was added in significant excess to a thiol-modified siRNA (as prepared in the previous siRNA methods section) and left at 4 °C for 3 h. The modified siRNA was then separated from free Azido-PEG3-Maleimide by SEC using an ENrich SEC 70 column (7801070 BioRad, Hercules, CA, USA) on a BioRad NGC Quest 10 Plus (7880003). Fractions containing the DBCO-sortagged antibody and azide-modified siRNA were concentrated using Pierce or Amicon concentrator columns (3K, 10K, or 30K MWCO). The purified and concentrated DBCO-modified antibody and azide-modified siRNA were then mixed overnight at 4 °C.

## 3. Results

### 3.1. Secreted Expression of Sortase A Δ59 Variant Srt4M in Expi293

Sortase A is used as a versatile bioconjugation reagent for the site-specific attachment of oligoglycine-containing molecules to an LPXTG amino acid sequence (sortase tag) present within another protein in a process referred to as sortagging [51,52]. As a protein of bacterial origin, the expression of sortase A is typically achieved in *E. coli*; however, production in typical strains inevitably results in lipopolysaccharide (LPS, endotoxin) contamination, complicating further research and clinical applications [53,54]. Therefore, we aimed to express sortase A Δ59 variant Srt4M [30,31], recently described to reach maximal ligation in as little as 15 min [30], in mammalian cells to eliminate endotoxin and therefore extend its use to LPS-free applications.

**Figure 1:**
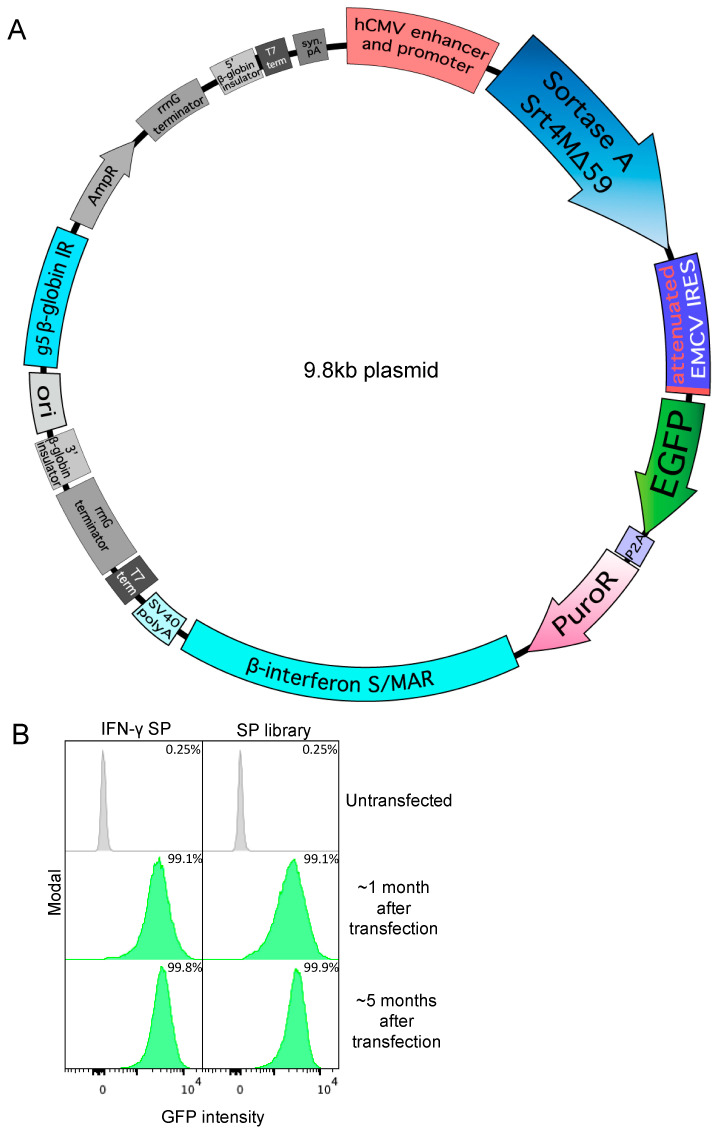
Specific signal peptides allow secretion of Srt4M in Expi293F cells. (**A**) Bicistronic Sortase A Srt4M Δ59 (Srt4M) plasmid map. Elements include, as indicated: human CMV (hCMV) promoter and enhancer, Srt4M, attenuated EMCV IRES, EGFP, P2A self-cleaving peptide, PuroR, β-interferon S/MAR, SV40 poly A (pA), and G5 β-globin replication initiation (IR). (**B**) Histograms show GFP expression intensity among un-transfected Expi293F cells or cells that were transfected with Srt4M plasmid as in A, using either the IFNγ signal peptide (SP) (left panel) or an SP library (right panel) (see text for details), and were then subject to selection with puromycin (see text) for 1 or 5 months, as indicated. Percentages denote the frequency of GFP^+^ cells in corresponding cultures, as indicated.

To express Srt4M, we designed an episomal vector with a single attenuated IRES [27,28] from encephalomyocarditis virus (EMCV) (bicistronic cassette) (Figure 1A). This bicistronic cassette contains an immediate early human CMV enhancer and promoter leading to the expression of Srt4M with a C-terminal His_6_ tag as the first cistron and linked by an attenuated IRES to an enhanced green fluorescent protein self-cleaving porcine teschovirus 2A peptide [55]-puromycin N-acetyltransferase (EGFP-P2A-PuroR) selection cistron further described in Methods. The self-cleaving P2A peptide allows EGFP and PuroR to separate during translation and function as independent proteins [56]. The complete expression vector also contains an S/MAR from human β-interferon [57] before the SV40 poly A signal, and the minimal β-globin IR, G5 [58], outside of the main expression cassette. Episomal elements, in combination with the linked attenuated IRES-mediated translation of the EGFP-P2A-PuroR cistron, allow the generation of stable Expi293F pools expressing a protein of interest by puromycin selection. It is also possible to monitor relative protein expression by EGFP. We included a vector backbone map listing the restriction enzymes in Figure S1A with expanded cloning details in Methods.

Sortase A Δ59 [31,59], a truncated version of the bacterial protein, has been engineered for secretion in bacteria [60], but to our knowledge has not been previously secreted in mammalian cells. The secretion of proteins in mammalian cells is a non-trivial task and attempts have been made to find “universal” signal peptides such as secrecon [32]. Güler-Gane et al. achieved the secreted expression of the proteins they studied using secrecon or secrecon with added N-terminal alanines but found secretion was especially unpredictable for proteins starting with cysteine, proline, tyrosine, or glutamine (sortase A Δ59 begins with glutamine). We first attempted to express Srt4M with secrecon and two alanines and were unable to achieve expression (as monitored by GFP) or generate a stable cell pool. This is consistent with the “all or nothing” phenomenon described by Güler-Gane et al., where inappropriate signal peptides achieve no expression. Instead of trying secrecon alone or secrecon plus a single alanine as previously described [32], we explored an alternative approach by using signal peptides from naturally secreted proteins with predicted signal peptide cleavage that exactly matched the +1/+2 amino acids of Srt4M (Gln-Ala).

We first tried the IFN-γ signal peptide from *Ailuropoda melanoleuca* (giant panda) as we reasoned that IFN-γ would be expressed highly in its natural context and the predicted +1/+2 amino acids perfectly matched those of Srt4M. Using the IFN-γ signal peptide, we achieved a cell pool expressing Srt4M after about 1 month of selection as indicated by the IRES-linked GFP expression measured by flow cytometry relative to an untransfected control (Figure 1B, left panel). The general gating strategy is shown in Figure S1B and applies to all subsequent histograms of GFP expression. In parallel, we also employed a signal peptide library approach using 17 different signal peptides from naturally secreted proteins that all perfectly matched the predicted +1/+2 cleavage (Gln-Ala) (Table S1). A mix of plasmids containing the library of 17 signal peptides for the expression of Srt4M was simultaneously transfected into Expi293F cells followed by selection, resulting in a viable GFP+ cell pool after the first month (Figure 1B, right panel). The dose of puromycin selection for IFN-γ and library transfections varied from 2 μg/mL to 100 μg/mL during the first month depending on cell density and viability as described in Methods. After the first month, puromycin concentration was kept at 30 μg/mL for the IFN-γ signal peptide Srt4M pool and 20 μg/mL for the signal peptide library Srt4M pool. In addition, between the first and second month, both pools were temporarily cryopreserved and stored in liquid nitrogen for 3 weeks before revival and continued selection. This selection was continued for an additional 4 months of culture. Both pools remained GFP+ during this period without an observable decrease in the mean GFP intensity, demonstrating stable transfection. (Figure 1B). Moreover, during this extended course of selection with puromycin, the width of the GFP distribution, measured by flow cytometry, narrowed, consistent with the cultures becoming more homogenous over time (Figure 1B). We examined the secretion of Srt4M from both the individual IFN-γ signal peptide stable pool as well as the signal peptide library stable pool by SDS-PAGE. We found similar Srt4M in the unpurified supernatants from both stably-transfected cell pools (Figure S1C) and after affinity purification, confirmed positive staining for His_6_ tag by western blot (Figure S1E).

In addition, after the 5-month selection period, we PCR amplified the signal peptide region from the stably-transfected signal peptide library Srt4M pool of cells and subjected the amplified linear fragments to next-gen nanopore sequencing (Plasmidsaurus), with results shown in Table S2. The signal peptide found at highest abundance was from Von Ebner gland protein 1 (*Rattus rattus*) followed by trefoil factor 1 (*Mus musculus*), both of which are signal peptides for proteins that are likely constitutively expressed as part of the mucosa throughout an organism’s life [61,62]. The next most abundant signal peptide was from an antibody light chain (*Homo sapiens*) followed by fibroblast growth factor binding protein 2 (*Homo Sapiens*) and lutropin subunit beta (*Oryctolagus cuniculus*). IFN-γ signal peptide was surprisingly not found, indicating that it could have been outcompeted over time by the pool of surviving cells or the numbers of individual cells with the IFN-γ signal peptide and possibly other signal peptides from the library were too low for detection at the sequencing depth used. We conclude that multiple signal peptides, including the IFN-γ signal peptide, could enable the successful secreted production of Srt4M. We anticipate these signal peptides could be useful for secreting other sortase A Δ59 variants.

The secreted expression of proteins in a mammalian host (as opposed to production in *E. coli* without natural glycosylation pathways) results in glycosylation if proteins have appropriate sequence motifs [63], thus resulting in multiple products with a range of molecular weights (MW) depending on the extent of glycosylation. We observed three separate bands by SDS-PAGE using Ni-NTA column-purified Srt4M (Figure S1C,D). The highest MW band appeared as most prevalent, the middle MW band was slightly less prevalent, and the lowest MW band was least prevalent (Figure S1C,D). After adding PNGase F (New England BioLabs, NEB) to purified Srt4M, all bands collapsed to the lowest MW band (Figure S1D). This result is consistent with the N-glycosylation of Srt4M when produced in mammalian cells and suggests the lowest MW band represents the species without N-glycosylation.

According to prediction using NetNGlyc 1.0 [63], the highest confidence N-glycosylation site is NETR starting at position 150 of Srt4M (9/9 agreement) (Figure S1F). Two other sites predicted with lower confidence are NESL (7/9 agreement) at position 107 and NISI starting at position 114 (5/9 agreement) (Figure S1F). We mapped the two asparagines predicted to be glycosylated with the highest confidence onto a previously reported crystal structure of sortase A and an LPETG peptide (PDB 1T2W) [64] (Figure S1G). Based on this structure, glycosylation at these sites seems unlikely to block enzymatic function as they do not directly overlap with LPETG peptide binding. However, the effects of glycosylation on the dynamics of a protein are hard to predict and it is difficult to make definitive conclusions without further experimentation. Nevertheless, we confirmed that the recombinant, mixed glycosylation-state Srt4M purified from Expi293 supernatants is functional by purifying it and utilizing it for the successful creation of a site-specific DAR1 antibody–siRNA conjugate as presented later in the text.

### 3.2. Stable Production of Antibodies with Heavy-Chain Tags

**Figure 2:**
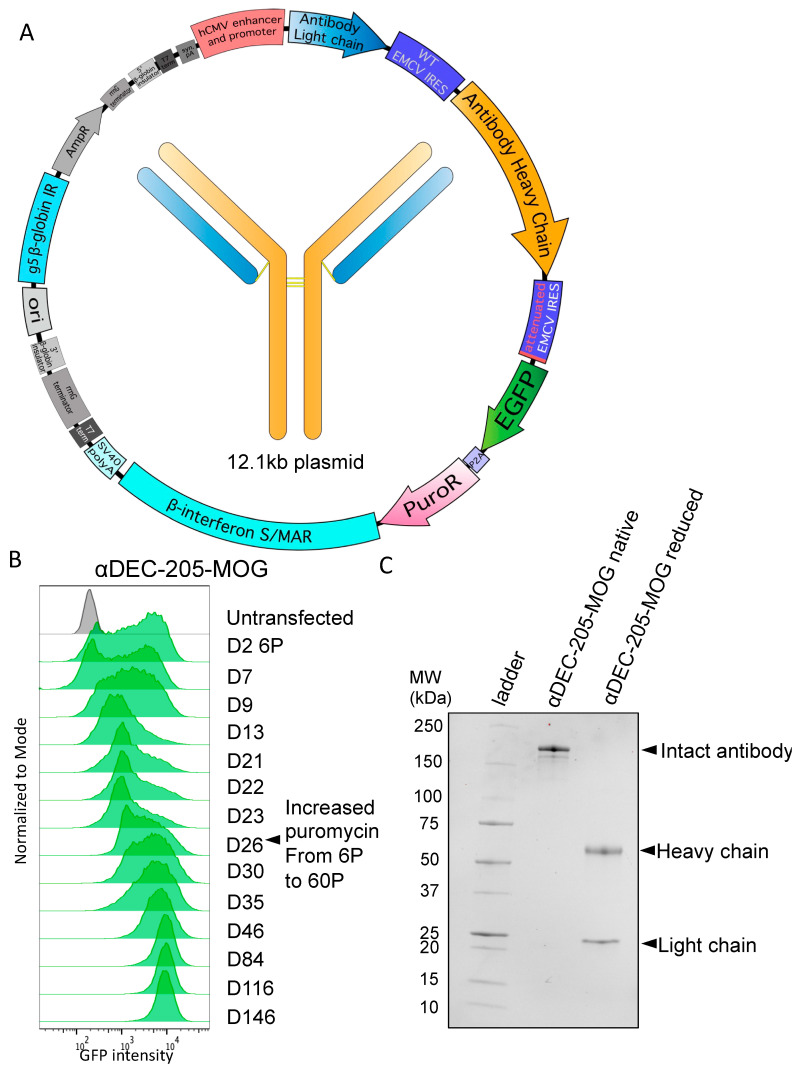
Generation of a stable cell pool expressing αDEC-205-MOG. (**A**) Tricistronic plasmid map for antibody expression. Elements include, as indicated: hCMV promoter and enhancer, antibody light chain, WT EMCV IRES, antibody heavy chain, attenuated EMCV IRES, EGFP, P2A self-cleaving peptide, PuroR, β-interferon S/MAR, SV40 pA, and G5 β-globin IR. (**B**) Cells were transfected with a tricistronic plasmid as in A that included heavy and light chains of αDEC-205-MOG. Histograms show GFP expression intensity over a time course ranging from 2 days to 146 days after transfection. Cells were initially selected at 6 μg/mL puromycin (6P), which was later increased to 60 µg/mL (60P) on D26 after transfection, as indicated. (**C**) SDS-PAGE gel showing protein G-purified αDEC-205-MOG with and without disulfide bond reduction performed before loading (reduced and native, respectively).

To enable production of a complete antibody molecule comprising heavy and light chains, we used a tricistronic cassette containing an immediate early CMV enhancer/promoter and an additional WT EMCV IRES (as described in Ho et al. in CHO cells [27,28]). The cassette is still linked to the attenuated EGFP-P2A-PuroR selection cistron as discussed above. Within this tricistronic cassette, the antibody light chain is the first cistron translated by cap-dependent translation. The second cistron is the antibody heavy chain, translated by WT EMCV IRES-mediated translation. Finally, EGFP-P2A-PuroR is the third cistron, translated by attenuated IRES-mediated translation. In addition to this tricistronic cassette, the complete expression vector, as shown in Figure 2A, contains the same S/MAR and IR elements already described earlier in the text for episomal maintenance in Expi293F cells. An expanded cloning protocol is described in Methods.

C-terminal antigen-fused recombinant antibodies based on the sequence of a chimeric αDEC-205 specific for mouse DEC-205 were first produced and extensively tested by Hawiger et al. [35]. Moreover, this specific design has been successfully used by numerous other investigators as we reviewed previously [37,65]. Therefore, we used this established design to examine the detailed kinetics of the process of stable pool generation using the vector with a tricistronic cassette. We used a sequence of αDEC-205, which contains myelin oligodendrocyte glycoprotein (MOG)_35–55_ peptide fused to the heavy chain C-terminus (αDEC-205-MOG), that was previously produced and characterized by Hawiger et al. [48] and subsequently other investigators, as we previously reviewed [37,65]. As seen in Figure 2B, an initial GFP^+^ pool was generated by selection with 6 µg/mL puromycin in approximately 23–26 days. To enable an accumulation of higher producing clones, the puromycin dose was then increased to 60 µg/mL. This resulted in a dramatic increase in mean GFP intensity that prevailed long-term and was stably maintained for at least 5 months of continuous culture with selection (Figure 2B). After completing the selection, we confirmed the production of the complete antibody that included both light and heavy chains by SDS-PAGE after protein G purification from supernatants (Figure 2C). Overall, we achieved successful stable expression of a C-terminally tagged antibody with the episomally replicating vector using a tricistronic cassette with attenuated expression of EGFP-P2A-PuroR.

### 3.3. Utilizing Inteins for Production of a Bispecific Antibody-like Molecule

For specific pairing of an antibody heavy chain with a protein–Fc fusion chain, we utilized complementary electrostatic steering mutations previously described by Wang et al. and established to result in 100% correct heavy chain pairing [66]. This approach involves point mutations E356K and D399K in one heavy chain CH3 domain and the complementary K439D and K409E mutations in the other heavy chain CH3 domain. These complementary mutations break the natural heavy chain symmetry and allow only charge-complementary pairing to occur [66]. We further combined this complementary charge approach based on specific mutations in different heavy chains with the SiMPl split intein–split puromycin system [41] to obtain a complete expression system for an immunoglobulin molecule with two different heavy chains, as described in further detail below.

**Figure 3:**
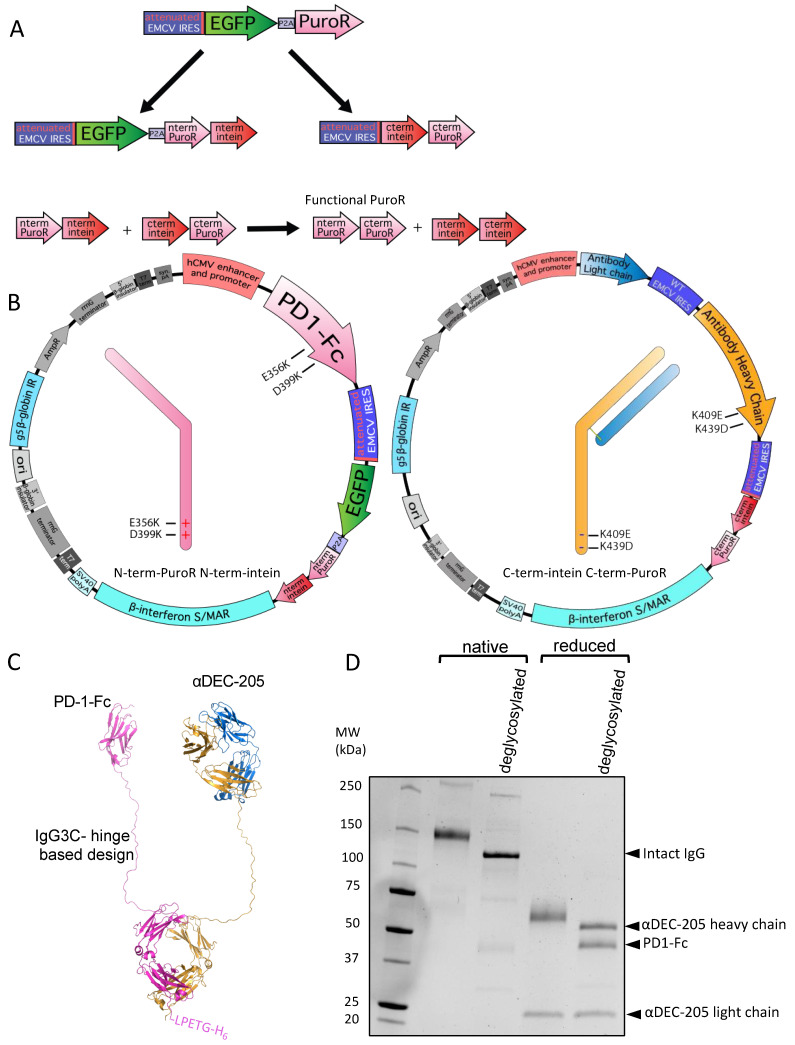
Split intein-mediated selection to produce a bispecific αDEC-205-PD-1-Fc molecule. (**A**) Diagram showing intein-mediated splitting of puromycin based on the SiMPl system. The EGFP-P2A-PuroR cistron is split into two cistrons, the EGFP-P2A-N-terminal-PuroR-N-terminal-intein cistron and the C-terminal-intein-C-terminal-PuroR cistron, both translated via an attenuated IRES as shown. When expressed together, both intein–PuroR fragments reform functional PuroR, as indicated. (**B**) Plasmid maps showing the linked intein–PuroR fragments with specific heavy chain mutations. E356K and D399K within PD-1-Fc (shown in pink) are linked to the N-terminal-PuroR-N-terminal-intein cistron (left). K409E and K439D within the αDEC-205 heavy chain (shown in orange) are linked to the C-terminal-intein-C-terminal-PuroR cistron (right). The αDEC-205 light chain is shown in blue. (**C**) Cartoon backbone representation diagram of an atomic model of αDEC-205-PD-1-Fc bispecific antibody-like molecule. The structure was first generated with Alphafold multimer 2.3.2 [68,69]. Rotations of each arm were then made in PyMOL (Schrödinger) for display. Disulfide bonds between the two heavy chains were added in BioLuminate (Schrödinger) and the molecule was minimized. Image generated using PyMOL (Schrödinger). (**D**) SDS-PAGE gel showing αDEC-205-PD-1-Fc after nickel column purification with and without disulfide bond reduction before loading (reduced and native, respectively) and, additionally, with and without deglycosylation mix II (NEB) treatment (deglycosylated as indicated). Arrows on side indicate bands of expected MW as described in the text representing the full bispecific molecule and individual heavy chains when deglycosylated.

Split inteins can be used to reconstitute, by the autocatalytic formation of a peptide bond, a protein that has been designed as two separate, dysfunctional parts [41,67]. Specifically, two dysfunctional fragments of PuroR protein, expressed under two separate cassettes, can be re-formed using split inteins [41]. We split the EGFP-P2A-PuroR cistron, originally present in a single expression cassette described in Figure 1A and Figure 2A, into two cistrons included in two separate expression cassettes. Specifically, we included EGFP-P2A-N-terminal-PuroR-N-terminal-intein in one cassette and C-terminal-intein-C-terminal-PuroR in another cassette (Figure 3A). In these two new expression cassettes, both intein–PuroR fragments are still expressed by attenuated IRES-mediated translation following the expression of the corresponding mutated heavy chain (Figure 3A,B). Therefore, for both intein–PuroR fragments to be expressed, to associate, and to splice together the functional PuroR, the expression of both complementary heavy chains is required. The individual expression cassettes can either be co-transfected as part of two separate plasmids (Figure S2A) or transfected together as part of a single plasmid with two promoters (Figure S2B), thus completing the episomal vector design. Expanded cloning details are included in Methods.

To demonstrate the utility of this system, we designed an αDEC-205-programmed cell death protein 1 (PD-1)-Fc bispecific construct (Figure 3B). The individual antigen-binding specificities of variable regions of recombinant αDEC-205 antibody expressed in 293-based cells have been previously established [35,37,65]. Mouse PD-1-Fc (BioLegend cat #764802) constructs are also well characterized and available from multiple commercial sources. We expressed the αDEC-205 light chain and heavy chain (with K409E and K439D mutations) from a tricistronic cassette with the C-terminal-intein-C-terminal-PuroR vector (Figure 3B right panel) and the mouse PD-1-Fc chain (with E356K and D399K mutations) was expressed from a bicistronic cassette with the N-terminal-PuroR-N-terminal-intein vector (Figure 3B left panel). We used a linker design (IgG3C-) similar to that pioneered by Bournazos et al. [49] for extended flexibility between both immunoglobulin arms, with a cartoon backbone representation of an atomic model shown in Figure 3C. We co-transfected both plasmids and achieved a stable pool in about 1.5–2 months. The PD-1-Fc chain also included a His_6_-tag that allowed the entire αDEC-205-PD-1-Fc molecule to be purified by Ni-NTA column. We purified αDEC-205-PD1-Fc using a Ni-NTA column and examined it by SDS-PAGE in native (non-reduced) and reduced forms with or without an additional deglycosylation step. We observed bands corresponding to the expected MW of 53.2 kDa for αDEC-205 heavy chain and 45.0 kDa for PD-1-Fc in the sample that was deglycosylated and run under reducing conditions. In addition, we observed a band consistent with an expected MW of 121.6 kDa for the entire αDEC-205-PD1-Fc molecule after its deglycosylation without disulfide reduction (native) (Figure 3D). In contrast, without the deglycosylation step, both native and reduced forms ran at higher than predicted MWs, consistent with the glycosylation of both heavy chains (Figure 3D). Overall, we conclude that intein-mediated selection coupled with complementary electrostatic steering mutations allows successful, stable production of a bispecific antibody-like molecule.

### 3.4. Split Inteins for Stable Production of Defined Multi-Tag Antibodies

**Figure 4:**
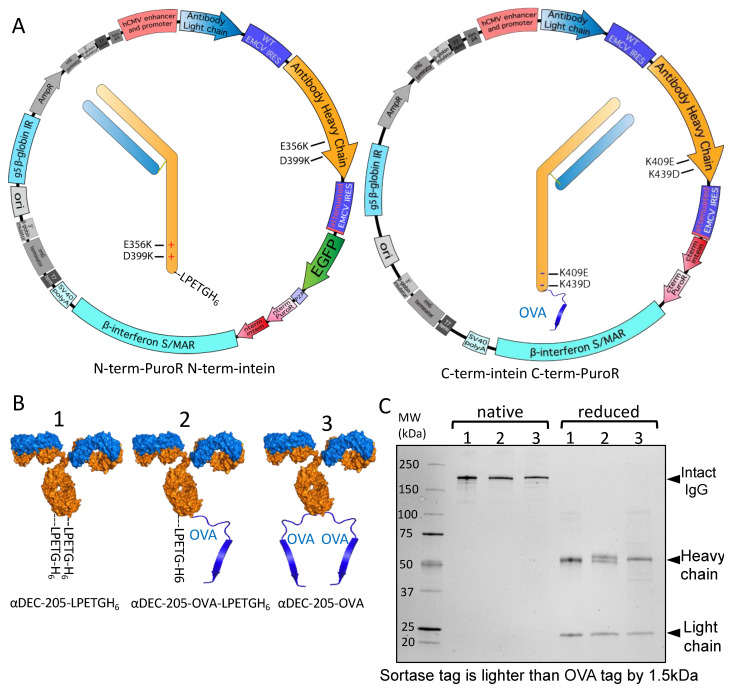
Split intein-mediated selection to produce antibodies with two different C-terminal tags. (**A**) Plasmid map showing coupling of LPETGH_6_ tag on one heavy chain (with E356K and D399K mutations) with EGFP-P2A-N-terminal-PuroR-N-terminal-intein (**left**) and coupling of OVA_323–339_ antigen on another heavy chain (with K409E and K439D mutations) with C-terminal-intein-C-terminal-PuroR (**right**). (**B**) Diagram of antibodies as numbered: 1—αDEC-205 antibody with a sortase tag and His_6_ tag (LPETGH_6_) on both chains. 2—αDEC-205 antibody with LPETGH_6_ on one chain and OVA_323–339_ antigen on the other chain. 3—αDEC-205 antibody with OVA_323–339_ antigen on both chains. (**C**) SDS-PAGE gel showing native and reduced antibodies from (**B**), as indicated by the numbers on top of the lanes. Native antibodies are loaded without disulfide bond reduction, while reduced samples have had disulfide bonds reduced before loading.

Intein-mediated selection coupled with complementary electrostatic steering mutations can also be applied to produce antibodies with distinct C-terminal modifications in their heavy chains. Following the strategy discussed in Figure 3 above, we designed αDEC-205 antibodies where one heavy chain (with K409E and K439D mutations) included an antigenic fragment from ovalbumin (OVA_323–339_) with a linker as previously published [47] while the other heavy chain (with E356K and D399K mutations) included the same linker followed by a sortase tag and His_6_ tag (LPETGH_6_). Transfecting these two cassettes linked to attenuated IRES-mediated expression of split intein–split puromycin fragments allows the creation of antibodies with an antigen and a single sortase attachment site (Figure 4A). We also separately generated stable Expi293F pools expressing heavy chain sequences without complementary mutations that have either OVA_323–339_ or LPETGH_6_ on both chains with the same linker. A depiction of these antibodies is shown in Figure 4B. Because the heavy chain with an LPETGH_6_ tag has an MW 1.5 kDa lighter than the MW of the heavy chain with OVA_323–339_, all three produced antibodies could be readily distinguished from each other by SDS-PAGE when disulfides were reduced (Figure 4C). Two bands of different MW but equal intensity are seen in the αDEC-205-OVA-LPETGH_6_ sample, as expected for this molecule that contains two different heavy chains. In contrast, and as expected, only a single corresponding heavy chain band is present in the case of αDEC-205-OVA or αDEC-205-LPETGH_6_ antibodies (Figure 4C). Overall, we conclude that by attenuated IRES-linked split intein-mediated selection, we successfully achieved the stable production of an antibody molecule that contains different C-terminally modified heavy chains, including a single sortase tag and a single antigen molecule.

### 3.5. Site-Specific DAR1 siRNA–Antibody Conjugate

**Figure 5:**
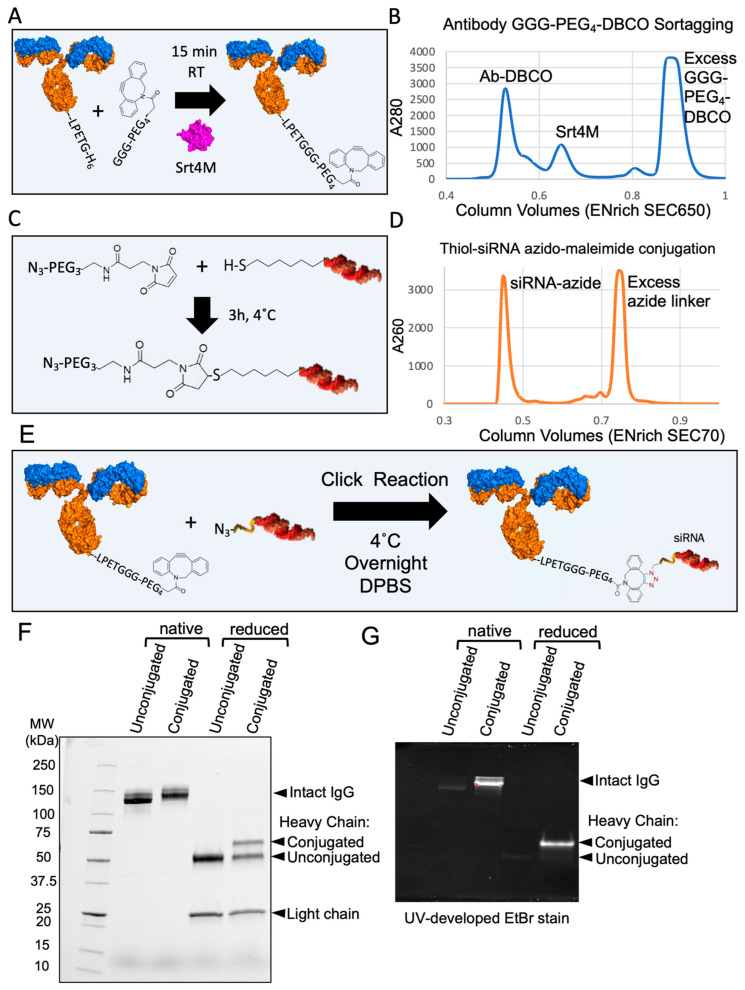
Creation of DAR1 antibody–siRNA conjugate by sortagging and click chemistry. (**A**) Diagram of DBCO-PEG_4_-GGG-mediated sortagging of antibody with a single sortase tag. GGG-PEG_4_-DBCO is sortagged onto the antibody using Srt4M as shown. (**B**) A280 chromatogram showing separation between antibody–DBCO conjugate, Srt4M, and free GGG-PEG_4_-DBCO linker using an ENrich SEC 650 column (BioRad). (**C**) Diagram of thiol-modified siRNA conjugation to azido-PEG_3_-maleimide. The maleimide group reacts with the free thiol on reduced siRNA to form the azide-modified siRNA conjugate as shown. (**D**) A260 chromatogram showing separation of siRNA–azide conjugate from excess azide-maleimide linker using an ENrich SEC 70 column (BioRad). (**E**) Diagram outlining formation of antibody–siRNA site-specific DAR1 conjugate using DBCO-azide-based click chemistry as shown. (**F**) 4–20% gradient SDS-PAGE gel of antibody–siRNA conjugate. Unconjugated and conjugated antibody with and without disulfide bond reduction before loading (reduced and native, respectively) are shown as indicated. (**G**) 7.5% SDS-PAGE gel of antibody–siRNA conjugate after gel staining with EtBr. Same sample loading order as in (**F**). Unconjugated and conjugated antibody with and without disulfide bond reduction before loading (reduced and native respectively) are shown as indicated.

To show the applicability of an antibody produced with a single sortase tag, we created a site-specific DAR1 siRNA–antibody conjugate. Based on the approach presented in Figure 4, we generated an antibody that contained a single sortase tag (LPETGH_6_) directly on the C-terminus of one heavy chain, without any linkers. Next, we made use of the recombinant Srt4M that we purified from Expi293 supernatants (Figure 1). An outline of the process of sortagging a GGG-PEG_4_-DBCO molecule to an antibody with a single LPETGH_6_ tag is shown in Figure 5A and additional details are included in Methods. Approximately 56 μg of Srt4M was added to 150 μg of antibody with excess GGG-PEG_4_-DBCO and 5 mM CaCl_2_ in a total volume of approximately 200 μL. After 15 min, EDTA was added to quench the reaction, and the antibody, Srt4M, and excess GGG-PEG_4_-DBCO were separated by size exclusion chromatography (SEC) (Figure 5B). A thiol-modified siRNA was conjugated to an azide-containing molecule using Azido-PEG_3_-Maleimide according to the manufacturer’s protocol (Figure 5C). The modified siRNA was also separated from free Azido-PEG_3_-Maleimide by SEC (Figure 5D). The purified DBCO-modified antibody and the purified azide-modified siRNA were then mixed overnight at 4 °C (Figure 5E) in a click chemistry reaction [42] and resolved by SDS-PAGE the following day (Figure 5F). A clear shift in MW (consistent with the ~14 kDa MW siRNA) was seen in the sample conjugated with siRNA without disulfide bond reduction. Analysis under reducing conditions further revealed the expected presence of both the siRNA-conjugated and unconjugated heavy chains (Figure 5F). This is an expected result as only one of the two heavy chains has a sortase tag and therefore, the ability to be conjugated. The similar relative intensities of the conjugated and unconjugated heavy chain bands indicate a high degree of conjugation of the DBCO-sortagged antibody. Additionally, we visualized the siRNA-conjugated antibody by resolving it on another gel that was imaged after incubation with ethidium bromide (EtBr), which intercalates double-stranded nucleotides like siRNA (Figure 5G). Despite a small amount of visible non-specific protein staining by EtBr, we observed a striking difference in band intensity corresponding to the siRNA-conjugated antibody and heavy chain, further confirming the presence of nucleic acid conjugation (Figure 5G). As expected, the conjugation of the siRNA site specifically to the antibody C-terminus did not affect the ability to bind to DEC-205, as shown in a direct competitive binding assay using PE-Cy7-conjugated anti-DEC-205 (NLDC-145) that shares the identical variable regions with recombinant anti-DEC-205 used for siRNA conjugation (Figure S3). Overall, we conclude that we successfully created a DAR1 siRNA–antibody conjugate by utilizing the key reagents that were all produced in Expi293F cells using our novel expression vectors and strategies.

## 4. Discussion

We present a comprehensive plasmid system useful for the stable long-term production of recombinant proteins, standard antibodies, modified antibodies, and bispecific antibody-like molecules. By combining episomal retention with a split selection of two separate protein expression cassettes, our approach represents a significant advancement for the stable production of complex molecules in Expi293F cells. We also demonstrated a novel approach to identifying signal peptides that allow the secretion of Srt4M in mammalian cells. Overall, our novel expression systems allowed us to produce recombinant proteins that we used to prepare a site-specific DAR1 antibody–siRNA conjugate.

We started with the general tricistronic cassette design as described by Ho et al. over a decade ago [27]. In this system, the expression of both antibody chains is linked to a single selectable marker expressed from an attenuated IRES. Critically, this tricistronic expression system allows greater protein productivity and increases the likelihood of the stable production of both chains over time [27,28,29]. However, this tricistronic approach based on a single antibiotic is limited to the stable expression of only one specific light chain and heavy chain. In this work, we present a major breakthrough for the stable production of more complex antibody molecules using two different tricistronic cassettes in combination with a split selectable marker. Specifically, each individual tricistronic cassette is linked to the expression of a split intein–split PuroR fusion protein, which on its own is not a functional selectable marker. It is only when two tricistronic cassettes are expressed together that both intein–PuroR fragments are expressed and can re-form the functional selectable marker. Therefore, this system permits the expression of up to four different protein chains linked to selection by a single selectable marker, unlocking a wide range of possibilities for the stable production of complex molecules, the full scope of which we have only just begun to explore in this paper.

We demonstrated the utility of paired tricistronic cassettes for achieving the stable production of antibodies with two different C-terminally modified heavy chains. We also showed an example of a tricistronic cassette paired with a bicistronic cassette encoding a protein–Fc fusion to stably produce an antibody–protein–Fc bispecific molecule. We anticipate that paired tricistronic cassettes should enable the production of classical bispecific antibodies, with two different light chain–heavy chain pairs. For example, highly specific approaches such as CrossMab^CH1-CL^ or similar methods that produce no incorrect light chain–heavy chain pairing [70,71,72] could work well in a tricistronic format where light chains are expressed slightly in excess to heavy chains [73].

While paired tricistronic cassettes have a clear advantage in enforcing the expression of four different protein chains by a single antibiotic, they are not strictly required to take advantage of attenuated IRES-mediated split intein selection. We expect that the vectors we generated would enable the expression of a single light chain and heavy chain from separate promoters as bicistronic cassettes linked to split selection with a single antibiotic. However, to adapt this approach to bispecific antibody expression, with each chain expressed under an independent promoter, would require either further splitting a single selection marker into four fragments or using two different split selection markers, such as split puromycin and split hygromycin, previously generated by Palanisamy et al. [41].

Biotherapeutic manufacturing requires yield optimization by a variety of methods including deriving and screening clonal lines and production by high-density (15–20 × 10^6^ cells/mL) fed-batch culture. In this work, we focused on the development of methods for the reliable production of proteins at the preclinical stage of development. We did not focus on absolute yield optimization and it is difficult to directly compare our approach to other approaches in the literature as yields are typically dependent on the particular construct being expressed. However, our stable pool of α-DEC-205-fused antigen-producing cells yields approximately 25–30 mg/L, which compares favorably to the transient transfections of similar α-DEC-205 C-terminally-tagged antibodies which yield 5–7 mg/L [74]. In addition, the inclusion of EGFP together with PuroR within the selection cistron provides the crucial ability to regularly monitor protein expression during the selection process as well as after the selection is complete. Furthermore, this design allows live cells to be selected by fluorescence-activated cell sorting. Although we did not perform extensive testing, it may be possible to sort for stable cells purely by EGFP expression. Therefore, our approach for the generation of stably transfected pools of cells may be useful as a starting point for preclinical protein production and could also find use in the production of biotherapeutics.

Our novel expression systems enabled us to make products that allowed the creation of a DAR1 site-specific antibody–siRNA conjugate. While the functional delivery of siRNA to cells is a complex topic and requires efficient endosomal escape [75], we anticipate the methods shown here could also be used to conjugate other nucleic acids, such as Toll-like receptor-activating adjuvants. The antibody-based delivery of various antigens to dendritic cells has now been well-established and broadly applied for modulating specific T-cell responses [37,65]. The current generation of dendritic-cell targeted antigen therapeutics in human cancer trials utilize a combination of many components including non-specifically delivered adjuvants such as Poly-ICLC and resiquimod [39,40]. We anticipate the approaches presented in this work could ultimately allow the delivery of nucleic acid-based adjuvants directly conjugated to engineered antibodies containing specific antigens. We also speculate that the use of other bond-forming enzymes [59,76,77,78,79], in addition to Srt4M, could make it possible to conjugate multiple antigens [80] along with an adjuvant to antibodies in a site-specific manner. These approaches could enable the next generation of dendritic cell-targeted antigen and neoantigen cancer therapeutics.

In closing, we present a robust and comprehensive expression system in Expi293F cells based on episomal replication with protein expression directly linked to selection. We use this system to reliably produce recombinant proteins and engineered antibodies in stably-transfected cells. We also demonstrated the applicability of the produced reagents by making a site-specific antibody–nucleic acid conjugate. Overall, these novel approaches and methods are likely to be of very high interest for research, preclinical, and industrial applications.

## Data Availability

The data presented in this study are available on request from the corresponding author. The plasmid backbones are available from Addgene as described in Section 2.

## Supplementary Materials

The following supporting information can be downloaded at: https://www.mdpi.com/article/10.3390/antib13010018/s1, Figure S1. EGFP-P2A-PuroR vector backbone and characterization of Srt4M; Figure S2. Split intein vector backbone maps; Figure S3. Competitive DEC-205 staining of MutuDC cell line pre-treated with siRNA-conjugated α-DEC-205 antibody; Table S1. Signal peptide library of all peptides used that had predicted +1/+2 QA cleavage residues matching those of Srt4M Δ59; Table S2. Signal peptides that were found after nanopore sequencing of PCR amplified DNA from cells selected with 20 ug/mL of puromycin for over 5 months.
