# Supplementary material for: Episomal Vectors for Stable Production of Recombinant Proteins and Engineered Antibodies
Source: Antibodies (Basel). 2024 Mar 11;13(1):18. doi: 10.3390/antib13010018 (PMC10967652; doi:10.3390/antib13010018)
Supplement: Supplementary file 1 [file antibodies-13-00018-s001.zip › antibodies-2879012-supplementary.pdf]

Table S1. Signal peptide library of all peptides used that had predicted +1/+2 QA cleavage residues matching those of Srt4M Δ59.

Signal Peptide (SP) Sequence	Protein Name	Species	Uniprot Accession	SP Length	Size (w/ SP)
MNYTSYILAFQLCVILCSSGYYC	IFN-γ	Ailuropoda melanoleuca	Q4ZH68	23	166
MASGVTITLAIAIFALEINA	Complement component C9	Rattus norvegicus	Q62930	20	554
MEHKVICVLAVVLMLAFGSLA	Trefoil Factor 1	Mus musculus	Q08423	21	87
MLAEWGACLLLAVALLGPGLQA	CUB domain contaning protein 2	Homo sapiens	Q5VXM1	22	449
MILSLLFSLGGPLGWGLLGAWA	Multimerin-2	Homo sapiens	Q9H8L6	22	949
MKALPALPLMLMLLSMPPPCAP	Microfibril-associated glycoprotein 4	Mus Musculus	Q9D1H9	22	257
MRLLVLAALLTVGAG	Phospholipase A2	Bos taurus	P00593	15	145
MKALLLTFGLSLLAALQA	Von Ebner gland protein 1	Ratus rattus	P20289	18	177
MGTLQGLLLWLLLGTGGA	lutropin subunit beta	Oryctolagus cuniculus	Q6IY74	18	141
MKFVPCLLLVTLSCLGTLG	fibroblast growth factor binding protein 2	Homo Sapiens	Q9BYJ0	19	223
MAWTPLLLLFLSHCTGSLS	Immunoglobulin lambda variable 5-45	Homo sapiens	A0A087WSX0	19	123
MAWTPLFLFLLTCCPGSNS	Immunoglobulin lambda variable 7-46	Homo sapiens	A0A075B6I9	19	117
MVSVPTTWCSVALALLVALHEGKG	Endothelin-2	Homo sapiens	P20800	24	178
MSGIGWQTLSLSLGLVLSILNKVAP	Slit homolog 2	Mus musculus	Q9R1B9	25	1521
MPGIKRILTVTILALCLPSPGNA	Fibulin-5	Homo sapiens	Q9UBX5	23	448
MVMLLLLLSALAGLFGAAEG	Apolipoprotein D	Homo sapiens	P05090	20	189
METQRASLCLGRWSLWLLLLALVVPSASA	Prophenin-2	Sus scrofa	P51525	29	228

Table S2. Signal peptides that were found after nanopore sequencing of PCR amplified DNA from cells selected with 20 ug/mL of puromycin for over 5 months.

Sequence	Protein	Species	# of reads
MKALLLTFGLSLLAALQA	Von Ebner gland protein 1	Ratus rattus	27
MEHKVICVLAVVLMLAFGSLA	trefoil factor 1	Mus Musculus	10
MAWTPLFLFLLTCCPGSNS	Immunoglobulin lambda variable 7-46	Homo Sapiens	6
MKFVPCLLLVTLSCLGTLG	fibroblast growth factor binding protein 2	Homo Sapiens	2
MGTLQGLLLWLLLGTGGA	lutropin subunit beta	Oryctolagus cuniculus	2

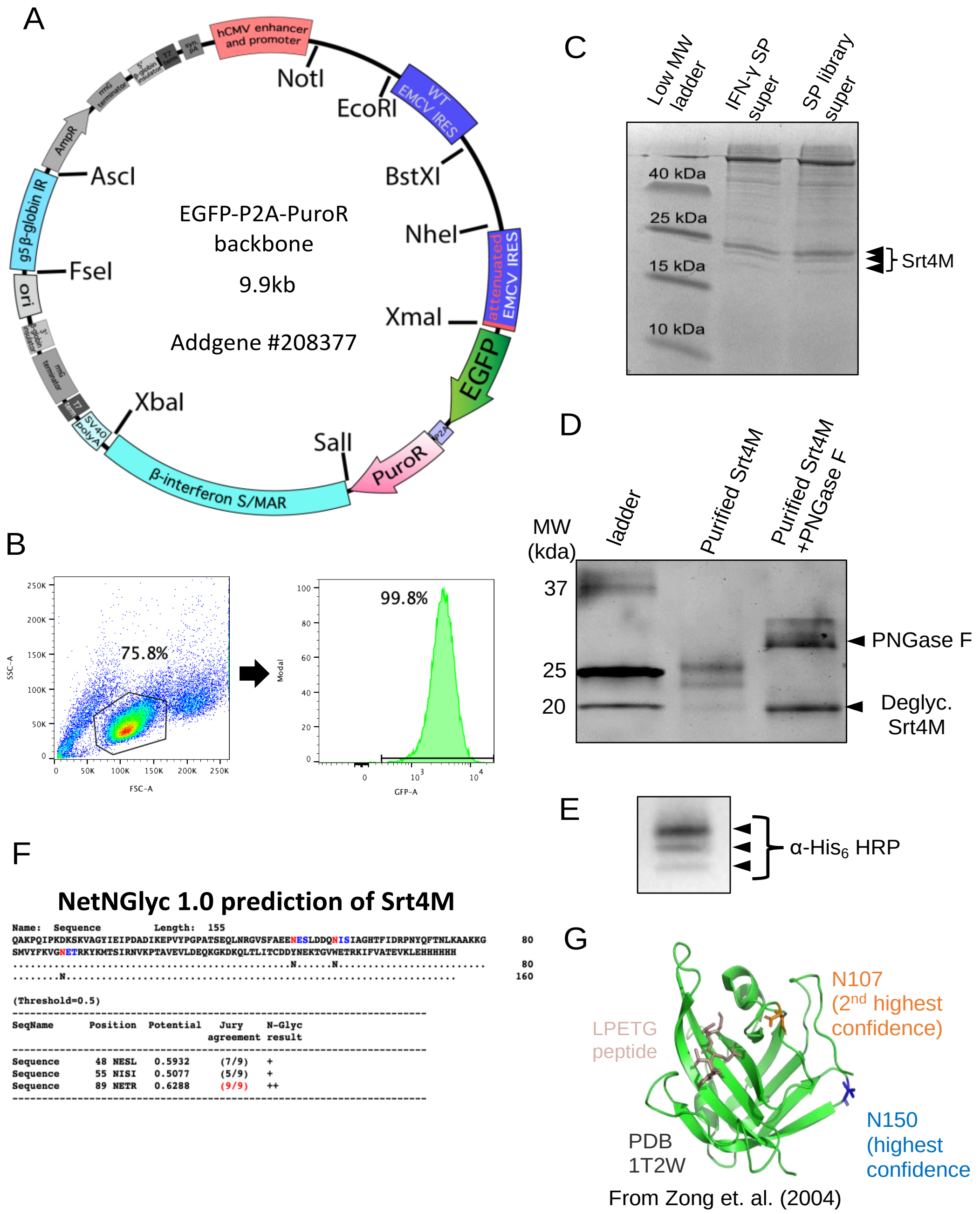

Figure S1. EGFP-P2A-PuroR vector backbone and characterization of Srt4M. **A.** EGFP-P2A-PuroR plasmid vector backbone with relevant unique restriction sites indicated. **B.** Gating strategy used to generate histograms of GFP expression intensity. **C.** Supernatants from stably-transfected Expi293 cell pools from B after 4-5 months of selection were resolved on an SDS-PAGE gel and are shown as indicated. **D.** SDS-PAGE gel showing Srt4M from the SP library pool of stably-transfected Expi293 cells. Nickel column-purified Srt4M with and without treatment with PNGase F deglycosylation is shown as indicated. **E.** Western blot staining for His_6 tag from purified Srt4M using HRP anti-His tag antibody (BioLegend 652503) developed using ChemiDoc with chemiluminescent substrate (BioRad). **F.** Output from NetNGlyc - 1.0 prediction [63] of N-glycosylation sites in Srt4M. **G.** Sortase A crystal structure (PDB: 1T2W, Zong et al 2004 [64]) with two highest confidence predicted glycosylation sites mapped in orange (N107) and blue (N150). The LPETG peptide is also shown in clam shell color. Image generated using PyMOL (Schrödinger).

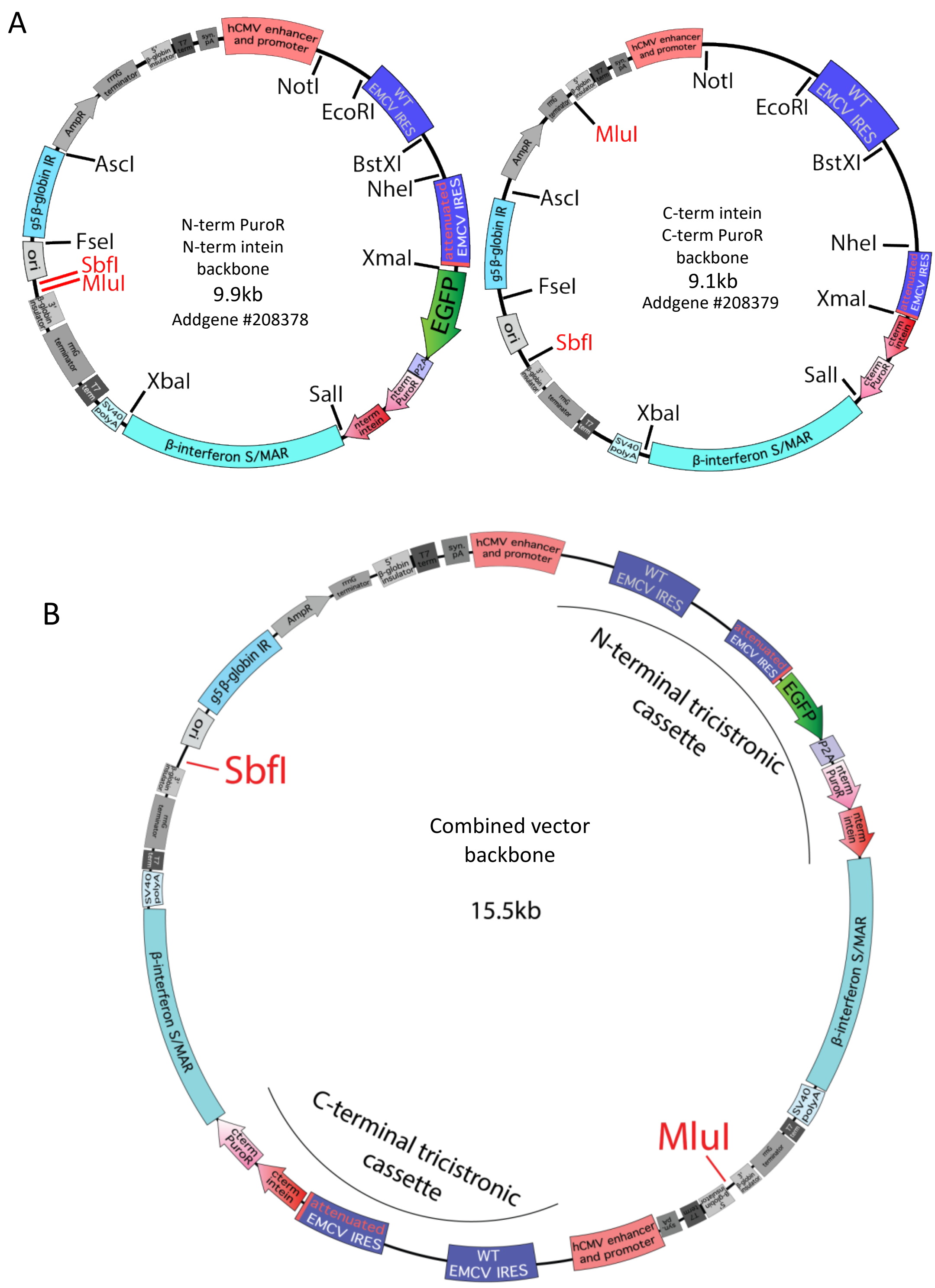

Figure S2. Split intein vector backbone maps. **A.** N-terminal PuroR N-terminal intein vector backbone with relevant unique restriction sites indicated (left). C-terminal intein C-terminal PuroR vector backbone with relevant unique restriction sites (right). SbfI and MluI sites are shown in red to highlight the different locations between the N-terminal and C-terminal plasmids whereas all other restriction enzyme sites are in identical locations in both plasmids. **B.** A combined vector containing both tricistronic cassettes linked to N- and C-terminal intein-puromycin cistrons.

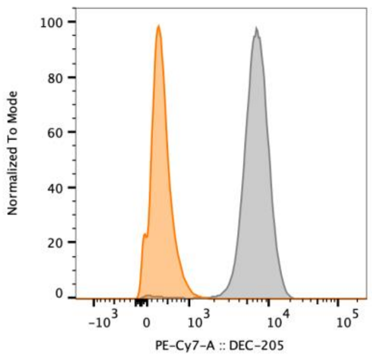

Figure S3 Competitive DEC-205 staining of MutuDC cell line pre-treated with siRNA-conjugated α-DEC-205 antibody. MutuDC were pre-treated (orange) or not pre-treated (gray) with α-DEC-205-siRNA at 5 μg/ml for 10 mins and subsequently stained with anti-mouse DEC-205 PE-Cy7 (BioLegend 138209) following the manufacture's protocol. The overlaid histogram shows PE-Cy7 intensity as indicated.
